# 100K Pathogen Genome Project

**DOI:** 10.1128/genomeA.00594-17

**Published:** 2017-07-13

**Authors:** Bart C. Weimer

**Affiliations:** Department of Population Health and Reproduction, School of Veterinary Medicine, UC Davis, Davis, California, USA

## Abstract

The 100K Pathogen Genome Project is producing draft and closed genome sequences from diverse pathogens. This project expanded globally to include a snapshot of global bacterial genome diversity. The genomes form a sequence database that has a variety of uses from systematics to public health.

## COMMENTARY

Whole-genome sequencing (WGS) has accelerated in recent years to produce over 300,000 public genomes. This progress has been, in part, fueled by lower-cost sequencing but more directly motivated by using WGS for food and public health applications. Early efforts to coordinate WGS began with the Lactic Acid Bacteria Consortium (1) that resulted in the reclassification of lactic acid bacteria (LAB) (2–5) and the release of genomes of *Lactococcus*, *Lactobacillus*, *Brevibacterium*, and *Bifidobacterium*. The 100K Pathogen Genome Project (http://100kgenomes.org; BioProject PRJNA186441) was established as an expansion of WGS for use in host-microbe interactions, public health, and genome ecology. The overall goal of the 100K Pathogen Genome Project is to produce high-quality draft genomes, as well as closed genomes of a variety of pathogens from food, animal disease, human disease, wildlife, and environmental reservoirs of those pathogens. It is also being used to inform accurate bacterial identification in metagenomic projects in food safety, where identification accuracy is of utmost importance.

To date, the 100K Pathogen Genome Project has released genomes from *Campylobacter* (6–11), *Shigella* (12), *Salmonella* (13–16), *Listeria* (6, 17), *Helicobacter* (18), and *Vibrio* (19) species, and more are in progress. The study by Kong et al. (15) is the largest release to date and is for *Salmonella*, with over 1,100 draft genomes from 185 serotypes and 130 untypeable isolates. The 100K Pathogen Genome Project uses standardized methods (20–26) to produce genomes with the number of contigs usually <300, and often under 50, with 50 to 100× coverage. Genomes of this quality allow confident measurement of diversity and functional characteristics; initial examples of this are represented in work by Chen et al. (27), who used closed *Salmonella* genomes to provide computational methods to estimate the epigenetic modification methylation status on a population scale as a link to possible function and *Listeria* in an attempt to link methylation status to virulence and risk (6). Initial examination of a variety of genomes provides insights into genome flexibility and rapid evolution on a microbial population scale that was inaccessible previously (28). Realizing that genome diversity is important for identification and functional capability, a global network of participants for the 100K Pathogen Genome Project was established with China, South Korea, and Mexico, with additional internationalization coprojects under way.
